# Intratracheally administered titanium dioxide or carbon black nanoparticles do not aggravate elastase-induced pulmonary emphysema in rats

**DOI:** 10.1186/1471-2466-12-38

**Published:** 2012-07-31

**Authors:** Agnès Roulet, Lucie Armand, Maylis Dagouassat, Françoise Rogerieux, Angélique Simon-Deckers, Esther Belade, Jeanne Tran Van Nhieu, Sophie Lanone, Jean-Claude Pairon, Ghislaine Lacroix, Jorge Boczkowski

**Affiliations:** 1 Inserm U955, Equipe 4, Créteil 94000, France; 2 Université Paris Est, Faculté de Médecine, Créteil 94000, France; 3 Institut National de l’Environnement Industriel et des Risques (INERIS), Verneuil en Halatte, France; 4 AP-HP, Hôpital Henri Mondor, Département de pathologie, Créteil, 94010, France; 5 Centre Hospitalier Intercommunal, Service de pneumologie et pathologie professionnelle, Créteil, 94000, France; 6 AP-HP, Hôpital Henri Mondor, Service de Physiologie Explorations Fonctionnelles, Créteil, 94000, France

**Keywords:** COPD, Occupational medicine, Particles, Toxicity

## Abstract

**Background:**

Titanium dioxide (TiO_2_) and carbon black (CB) nanoparticles (NPs) have biological effects that could aggravate pulmonary emphysema. The aim of this study was to evaluate whether pulmonary administration of TiO_2_ or CB NPs in rats could induce and/or aggravate elastase-induced emphysema, and to investigate the underlying molecular mechanisms.

**Methods:**

On day 1, Sprague-Dawley rats were intratracheally instilled with 25 U kg^−1^ pancreatic porcine elastase or saline. On day 7, they received an intratracheal instillation of TiO_2_ or CB (at 100 and 500 μg) dispersed in bovine serum albumin or bovine serum albumin alone. Animals were sacrificed at days 8 or 21, and bronchoalveolar lavage (BAL) cellularity, histological analysis of inflammation and emphysema, and lung mRNA expression of heme oxygenase-1 (HO-1), interleukin-1β (IL-1β), macrophage inflammatory protein-2, monocyte chemotactic protein-1, and matrix metalloprotease (MMP)-1, and -12 were measured. In addition, pulmonary MMP-12 expression was also analyzed at the protein level by immunohistochemistry.

**Results:**

TiO_2_ NPs *per se* did not modify the parameters investigated, but CB NPs increased perivascular/peribronchial infiltration, and macrophage MMP-12 expression, without inducing emphysema. Elastase administration increased BAL cellularity, histological inflammation, HO-1, IL-1β and macrophage MMP-12 expression and induced emphysema. Exposure to TiO_2_ NPs did not modify pulmonary responses to elastase, but exposure to CB NPs aggravated elastase-induced histological inflammation without aggravating emphysema.

**Conclusions:**

TiO_2_ and CB NPs did not aggravate elastase-induced emphysema. However, CB NPs induced histological inflammation and MMP-12 mRNA and protein expression in macrophages.

## Background

Pulmonary emphysema is a chronic degenerative lung disease characterized by an imbalance in alveolar destruction and repair that results in progressive destruction of pulmonary alveoli and chronic respiratory failure [1]. Pulmonary emphysema is one of the components of chronic obstructive pulmonary disease (COPD), a frequent condition with a worldwide prevalence of more than 10% in men older than 40 years. The prevalence and mortality of COPD are predicted to further increase in the next decades [2]. Different processes are involved in alveolar destruction in emphysema, particularly excess of protease expression and activity in the lung. In addition to neutrophil elastase, macrophage matrix metalloproteinase (MMP)-1 and -12 have a critical role in pulmonary emphysema (see reference [3] for review).

Nanotechnology is a continually developing field. Reducing a particle’s size to the nanometric dimension can greatly modify its properties for applications in numerous fields such as electronics, materials or health. However, concerns have arisen about the possible effects of these manufactured nanoparticles (NPs) on the health of users and/or workers manipulating them [4]. Indeed, the adverse health effects and toxicity of NPs are being increasingly evaluated (see for example reference [5]).

Titanium dioxide (TiO_2_) is one of the most abundantly produced NPs [6]. Because of its optical, photocatalytic and self-cleaning properties, it is widely used in paints, pigments, and cosmetics. Nevertheless, exposure to TiO_2_ NPs has various biological effects [7]. Respiratory exposure to TiO_2_ NPs in animals was found associated with pulmonary inflammation [8,9], parenchymal remodeling [10], including emphysema [11], and cancer [12]. TiO_2_ NPs exposure has been linked to an increase in MMP expression: MMP-12 in murine macrophages [13] and MMP-7, -9 and -10 in keratinocytes [14]. Moreover, TiO_2_ NPs induced the expression of interleukin-1β (IL-1β) [9], a strong inducer of MMP-1 [15]. Therefore, we hypothesized that exposure to TiO_2_ NPs could lead to and/or aggravate pre-existing pulmonary emphysema, given the roles of MMP-1 and MMP-12 in the pathophysiology of the disease [1]. The potential aggravation of pre-existing emphysema by TiO_2_ NPs is important because of the frequency of COPD and the possibility of patients with COPD having been exposed to TiO_2_ NPs.

Carbon black (CB) NPs are also abundantly produced [6]. Exposure to CB NPs can induce cell apoptosis [16], inflammation [17], or aggravate already existing pathologic conditions such as elastase-induced lung injury [18]. However, the comparative effects of CB and TiO_2_ NPs on emphysema induction or aggravation have not been examined yet. This is an important point because the effects of NPs depend in part on their chemical nature, and thus we can expect differences in emphysema generation and/or aggravation related to the chemical nature of the considered NP.

Therefore, we aimed to assess whether pulmonary administration of TiO_2_ or CB NPs in rats could induce and/or aggravate emphysema and to investigate the molecular mechanisms involved. We used a well-established elastase-induced pulmonary emphysema rat model, which reproduces key phenomena occurring in alveolar walls of patients with emphysema [19-21]. NPs were administered 7 days after elastase instillation, to assess the effects on the development of already constituted but still progressing emphysema [21]. We examined animals at 2 time-points and exposed them to 2 doses of TiO_2_ or CB NPs. We also characterized the physicochemical properties of NPs in terms of size, morphologic features, surface area, degree of dispersion and endotoxin content, as factors able to influence their biological properties [5,22].

## Methods

### Chemicals

Anatase TiO_2_ NPs (reference 637264) were purchased from Sigma Aldrich (St Louis, MO, USA). CB NPs (reference Carbon Black FW 2) were purchased from Degussa (Dusseldorf, Germany). In order to disperse NPs using a biological agent, TiO_2_ and CB NPs were suspended in 0.5 mg/ml bovine serum albumin (BSA, Sigma Aldrich, St Louis, MO, USA) as described previously [23].

### Characterization of NPs

Specific surface area was measured using Brunauer Emmett Teller (BET) adsorption isotherms of nitrogen at 77 K. Particle Size Distribution (PSD) function and zeta potential were determined in saline + 0.5 mg/ml BSA using a Zetasizer Nano S (Malvern Instruments Ltd, Worcestershire, UK). Transmission electronic microscopy (TEM) was performed in NPs suspensions filtered and dried overnight on 200-mesh copper grids. Representative images of the samples were taken using a JEOL 1200 EXII transmission electron microscope (OXFORD LINK ISIS 300) and analyzed using an image analyzer (SAISAM Software, Microvision). NPs endotoxin content was examined using the Limulus Amebocyte Lysate (LAL) kit QCL-1000 (Lonza, Basel, Switzerland) according to the manufacturer’s instructions.

### Animals

240 male Sprague-Dawley rats aged 6 to 8 weeks were purchased from Janvier (Le Genest Saint Isle, France). The rats were kept in a conventional animal facility and housed in positive-pressure air-conditioned units (22 °C, 60% relative humidity) on a 12 h:12 h light/dark cycle. They had *ad libitum* access to food and water. The experimental protocol has been approved by the ethical committee for animal research of the INERIS (*“Institut National de l’Environnement Industriel et des Risques”,* French National Institute of Industrial Environment and Risks).

### Intratracheal instillation studies

All animals were anaesthetized by intramuscular injection of a mixture of ketamine (1.6 mg, Merial, Lyon, France) and xylazine (300 μg, Bayer, Puteaux, France). The trachea was surgically exposed, and sterile saline solution or 25 U kg^−1^ porcine pancreatic elastase (Elastin Products, Owensville, MO) diluted in sterile saline solution was instilled, in a volume of 150 μl. Seven days after elastase or saline instillation (D7), rats underwent intratracheal instillation with BSA (0.5 mg/ml) or TiO_2_ or CB NPs (100 or 500 μg/rat). Half of the rats were sacrificed the day after NPs instillation (day 8 [D8] after elastase), and the other half 14 days after NPs instillation (day 21 [D21] after elastase). Therefore, 6 groups of animals (n = 10 in each one) were constituted per sacrifice time (D8 and D21, respectively) and per dose of NPs (100 μg and 500 μg, respectively): saline + BSA (S-BSA), saline + TiO_2_ (S-TiO_2_), saline + CB (S-CB), elastase + SA (E-BSA), elastase + TiO_2_ (E-TiO_2_), and elastase + CB (E-CB).

For each experimental condition, 2 groups (group A and group B) of rats were used, with 6 animals in group A and 4 in group B. Bronchoalveolar lavage (BAL) and Real-Time quantitative PCR (RT-qPCR) of the total lung were performed on rats included in group A. Histopathology and immunohistochemistry were performed in the lungs of rats included in group B. These lungs were fixed by airway instillation with 4% phosphate buffered paraformaldehyde at a pressure of 20 cm fluid column for 24 h, before being embedded in paraffin. Great-axis sagittal sections (5 μm) were performed in a systematic manner on the same part of the lung. Some sections were stained with hematoxylin and eosin (H&E) for histological studies; others were kept unstained for immunohistochemical analysis.

### Broncho alveolar lavage cellularity

BAL cellularity was analyzed in anesthetized animals as previously described [24].

### Histopathological analysis

We quantified histological markers of inflammation and emphysema by optical microscopy (Zeiss Axiophot, Carl Zeiss, Oberkochen, Germany). The inflammatory abnormalities studied were the following: alveolar inflammation, perivascular oedema and perivascular/peribronchial infiltration as described previously [25]. Analysis and quantification of the histopathological alterations were performed by 2 independent investigators, including an unrelated expert pathologist who was blinded from the experimental design and the treatments the animals received. From every animal, four to five sections were taken from different depths to give a representative appreciation of the whole lung. All histological alterations of the same type present on each section were photographed, and surrounded using the Zeiss Axiovision 40 software (Carl Zeiss), which gave the corresponding area for each alteration. In each section, a global alteration score of each inflammatory abnormality was calculated as the sum of the areas obtained. Median areas were determined from the different sections of the individual animals, and area values of the different experimental groups were calculated from the individual values.

Emphysema was quantified as described previously [24,26]. Briefly, the lungs were fixed with 2.5% glutaraldehyde at a transpleural pressure of 25 cmH_2_O for 3 h and held in 4% paraformaldehyde (Sigma). Great-axis sagittal sections (5 μm) of the left lung were cut in a systematic fashion and were stained with hematoxylin and eosin. Three black-and-white digital photomicrographs were acquired from the cranial, medial, and caudal regions of each slide at X40 magnification, excluding areas where large bronchi or vessels predominated, resulting in a total of 9 images per lung. Emphysema was then quantified by measurement of the mean chord length of alveoli [27,28] with Analysis software (Soft Imaging System, Münster, Germany) at a 5-μm interval. The analysis was performed in duplicate by two blinded observers (AR and LA). The mean chord length of alveoli was obtained by averaging those measurements.

### RT-qPCR

RT-qPCR in lung homogenates was performed to examine the mRNA expression of anti-oxidant, inflammatory and proteases genes as previously described [24]. Genes analyzed were heme oxygenase 1 (HO-1), interleukin 1β (IL-1β), macrophage inflammatory protein 2 (MIP-2), monocyte chemotactic protein 1 (MCP-1), MMP-1 and MMP-12. The primer sequences are given in Table1. Gene expression was normalized to that of hypoxanthine phosphoribosyltransferase (HPRT) as a housekeeping gene. 

**Table 1:** Primers used for real-time PCR

**Name**	**Genbank Accession Number**	**Sequences (5′- > 3′)**	**Product size (bp)**
HO-1	NM_012580.2	TGCTGACAGAGGAACACAAAGA	186
		CGGTCGCCAACAGGAAACT	
IL-1β	NM_031512.2	CTGTGACTCGTGGGATGATG	210
		GGGATTTTGTCGTTGCTTGT	
MCP-1	NM_031530	CCAGAAACCAGCCAACTCTCA	90
		TGTGAACAACAGGCCCAGAAG	
MIP-2	NM_053647.1	AGGATCGTCCAAAAGATACTGAACA	90
		TTGATTCTGCCCGTTGAGGTA	
MMP-1	NM_001134530.1	GCCAACAGGTGCAACAACAC	186
		GCATCAAGTTTACCTGGCAGATT	
MMP-12	NM_053963.2	CCCCAACACATTTCGTCTCTCT	90
		GGATTTGTCAAGGATGGGTTT	
HPRT	NM_012583.2	TTGGAAAGGGTGTTTATTCCTCAT	261
		ATCCAGCAGGTCAGCAAAGAA	

***Abbreviations: HO-1*heme oxygenase-1,*IL-1β*interleukin-1β,*MCP-1*macrophage inflammatory protein-2,*MIP-2*monocyte chemotactic protein-1,*MMP-1*matrix metalloprotease-1,*MMP-12*matrix metalloprotease-12,*HPRT*hypoxanthine phosphoribosyltransferase.**

### Immnunohistochemistry

Immunohistochemical staining was performed to detect the protein expression of MMP-12 (anti-MMP-12 antibody, Santa Cruz Biotechnology, Santa Cruz, CA; 1:100 dilution) and the macrophage marker CD68 (anti-CD68 antibody, Abcam, Cambridge, UK; dilution 1:200). Quantification was performed using ImageJ software [29]. Results were expressed as the percentage of stained cells versus total cells.

### Statistics

Data were expressed as median and interquartile range (IQR; 25^th^ and 75^th^ percentiles). Analysis was performed using GraphPad Prism 4.0 software (La Jolla, CA, USA). Comparisons between multiple groups were performed using Kruskall-Wallis non-parametric ANOVA followed by Mann-Whitney U test when a difference was detected. All comparisons were realized with correction for multiple comparisons. *p* < 0.05 was considered statistically significant.

## Results

### Characterization of NPs

The detailed physicochemical characterization of NPs (surface area, primary particle size, zeta potential and hydrodynamic diameter) is given in Table2**.** CB NPs had a higher specific surface area than TiO_2_ NPs (*p* < 0.05). The primary diameter of CB and TiO_2_ NPs was not statistically different. The zeta potential for CB and TiO_2_ NPs, used to indicate suspension stability, was about |20| mV, a commonly used limit above which the suspension is aggregated [30]. Indeed, PSD studies showed aggregation for both NPs, with a higher average hydrodynamic diameter of CB as compared to TiO_2_ NPs. However, as for the primary diameter, this difference was not statistically significant. 

**Table 2:** Nanoparticles characterization

**Nanoparticle**	**Crystal phase**	**Surface area (m²/g)**	**Average size (nm) (a)**	**Zeta potential (mV)**	**Hydrodynamic diameter (nm)**
CB	Amorphous	373 ± 18 *	23 ± 6	−21.4	613 ± 145
TiO_2_	Anatase	141 ± 6	12 ± 2	−16.9	451 ± 80

*:*p* < 0.05 vs TiO_**2**_**.**

**(a) From reference**[43].

***Abbreviations: CB* carbon black,*TiO***_***2***_**titanium dioxide.**

Data are mean ± standard error of mean.

No endotoxin was detected whatever NP (data not shown).

### BAL cellularity

**Figure 1:**
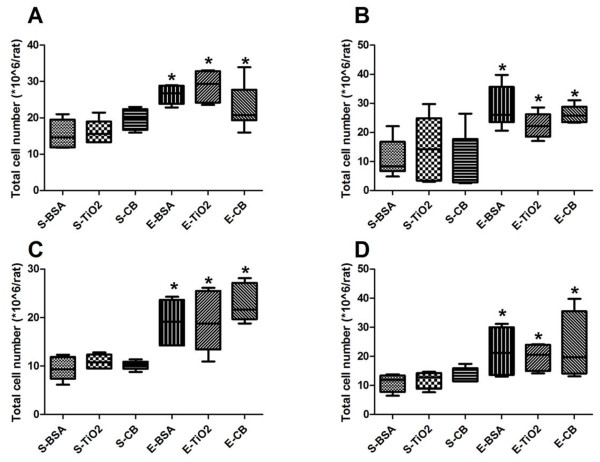
**Total cell number in BAL.** Quantification of BAL total cell number at D8 (**A**, **C**) and D21 (**B**, **D**) post elastase treatment, in presence of 100 μg (**A**, **B**) or 500 μg NPs (**C**, **D**). S, saline; E, elastase; BSA, bovine serum albumin. CB, carbon black. TiO_2_, titanium dioxide. *: *p* < 0.05 vs S-BSA. n = 6. Box plots show median and interquartile range (25^th^ and 75^th^ percentiles).

**Figure 2:**
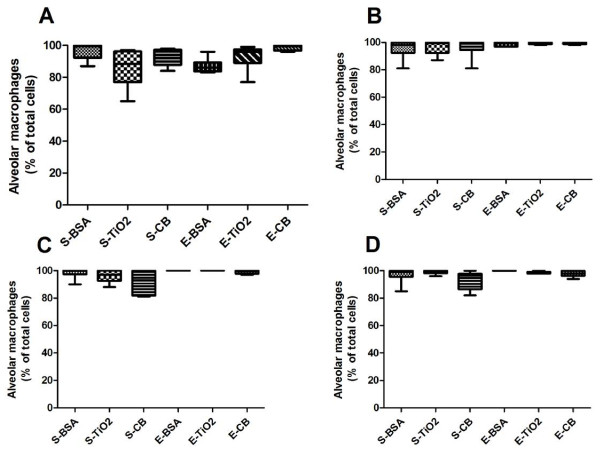
**Proportion of alveolar macrophages in BAL.** Quantification of the differential cell count in BAL at D8 (**A**, **C**) and D21 (**B**, **D**) post elastase treatment, in presence of 100 μg (**A**, **B**) or 500 μg (**C**, **D**) NPs. n = 6. Abbreviations are the same as in Figure1. Box plots show median and interquartile range (25^th^ and 75^th^ percentiles).

At D8 and D21, with saline treatment, NPs instillation (at both 100 and 500 μg/rat) did not modify BAL cellularity (Figure1). By contrast, in presence or absence of NPs, elastase induced a significant increase in total BAL cellularity as compared to saline treatment (*p* < 0.05, Figure1). Alveolar macrophages represented more than 80% of total cells in BAL, with no significant difference between groups (Figure2).

### Emphysema quantification

**Figure 3:**
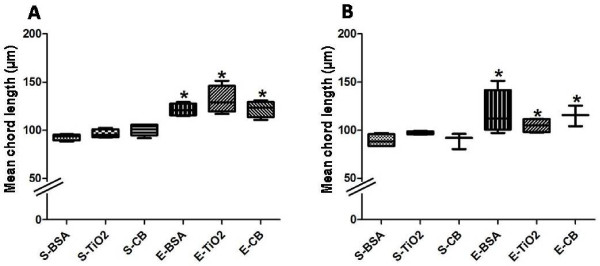
**Emphysema quantification.** Emphysema quantification assessed by measurement of the alveolar mean chord length at D21, in presence of 100 μg (**A**) or 500 μg (**B**) NPs. *: *p* < 0.05 vs S-BSA. n = 4. Abbreviations are the same as in Figure1. Box plots show median and interquartile range (25^th^ and 75^th^ percentiles).

Emphysema was quantified at D21 as described previously [24,26]. NPs did not induce emphysema *per se* (Figure3). As expected, mean airspace chord length was greater with elastase than saline treatment (*p* < 0.05, Figure3A, B), in absence or presence of whatever dose of NPs. 

Considering that the effects of NPs on BAL cellularity and emphysema were similar when the animals were exposed to 100 or 500 μg NPs, we used 500 μg NPs for the remaining studies.

### Histological analysis of inflammation

**Figure 4:**
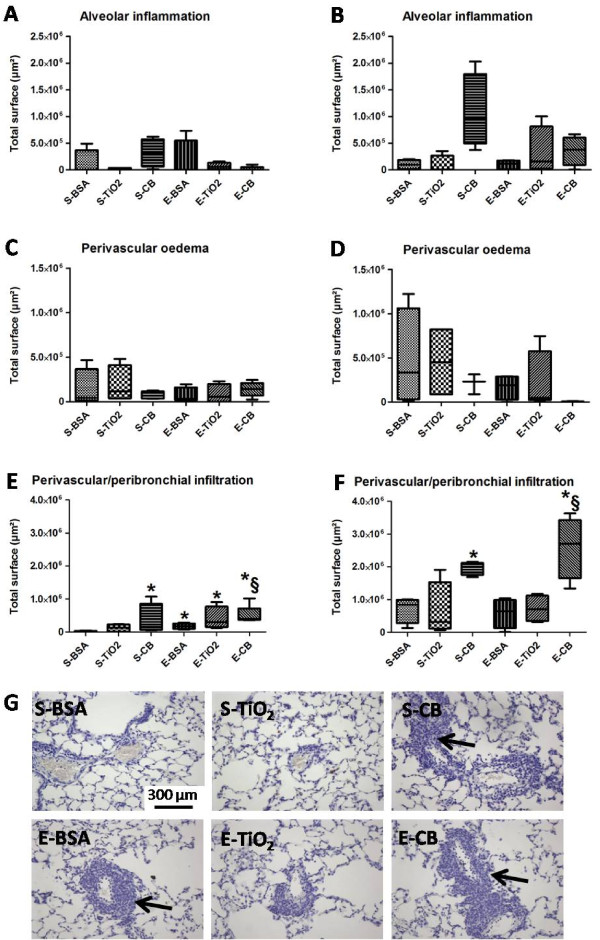
**Quantitative analysis of histological inflammation in animals receiving 500 μg NPs.** Histological inflammation was quantified in terms of alveolar inflammation (**A**, **B**), perivascular oedema (**C**, **D**) and perivascular/peribronchial infiltration (**E**, **F**) 8 days (**A**, **C**, **E**) or 21 days (**B**, **D**, **F**) after initial elastase administration in animals receiving 500 μg NPs. Data are expressed as the total surface of the histological slide presenting the histological feature. *: *p* < 0.05 vs S-BSA. §: *p* < 0.05 vs E-BSA. n = 4. Abbreviations are the same as in Figure1. Box plots show median and interquartile range (25^th^ and 75^th^ percentiles). Panel **G**: representative histological staining of perivascular/peribronchial infiltrates in rat lung at D21 in animals exposed to 500 μg NPs. Arrows point to perivascular infiltrates. Magnification x20.

At D8 and D21, rats receiving saline or elastase showed no alveolar inflammation nor perivascular oedema in absence or presence of NPs (Figure4A-4D).

At D8, with saline treatment, perivascular infiltration was greater in rats exposed to CB but not TiO_2_ NPs than BSA-treated animals (*p* < 0.05, Figure4E). Elastase treatment resulted in a significant increase in perivascular infiltration (*p* < 0.05, Figure4E). Moreover, exposure of elastase-treated rats to CB slightly but significantly increased this phenomenon. The infiltrates were mainly composed of lymphocytes, with also neutrophils and macrophages (Figure4G). Some infiltrates were also located peribronchially. A similar pattern was observed at D21 (Figure4F).

### Gene expression quantification

At D8, no induction of HO-1, IL-1β, MIP-2, MCP-1, MMP-1 or MMP-12 was observed after NP exposure alone, elastase instillation alone or elastase followed by NP exposure (data not shown).

**Figure 5:**
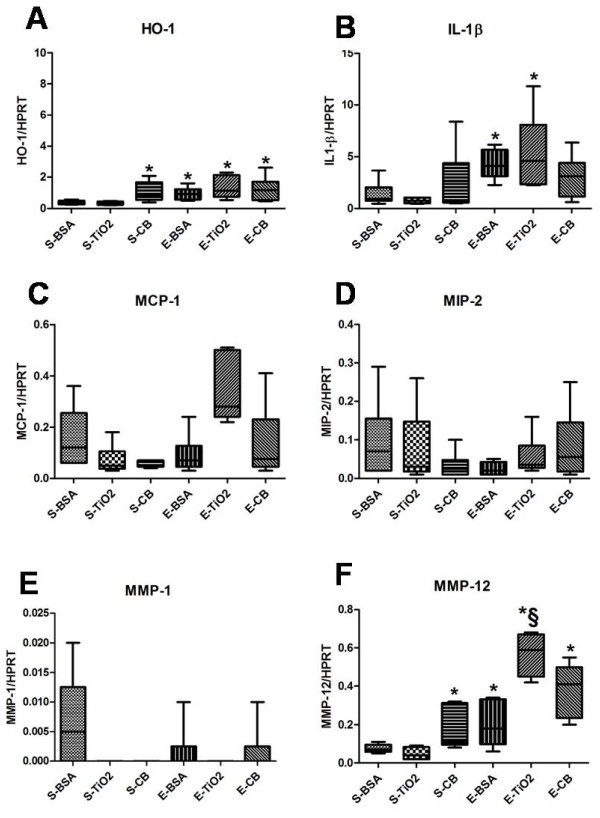
**Gene expression at D21 in animals receiving 500 μg NPs.** Quantification of mRNA expression of the different genes at D21 by RT-qPCR. Data are expressed as the ratio to HPRT as housekeeping gene. Rats were exposed to 500 μg NPs. *: *p* < 0.05 vs S-BSA. §: *p* < 0.05 vs E-BSA. n = 6. Abbreviations are the same as in Table1. Box plots show median and interquartile range (25^th^ and 75^th^ percentiles).

At D21, with saline treatment, the mRNA levels of HO-1 and MMP-12 were increased in rats exposed to CB but not TiO_2_ NPs (*p* < 0.05; Figure5A, F). With elastase treatment, the mRNA levels of HO-1, IL-1β and MMP-12 were higher than that with saline treatment (Figure5A, B and F); a similar pattern was observed in rats instilled with CB NPs. With elastase treatment, the mRNA level of MMP-12 was higher in rats with TiO_2_ exposure than BSA exposure (Figure5F).

### MMP-12 and CD68 immunohistochemical analysis

**Figure 6:**
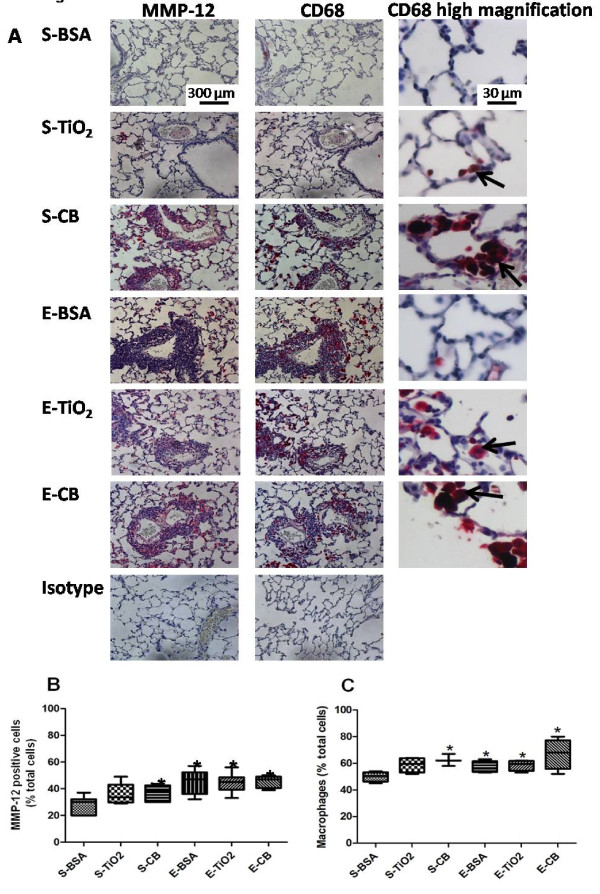
**Quantification of MMP-12 and CD68 immunostaining at D21 in animals receiving 500 μg NPs.** Localization and quantification of MMP-12 and CD68 on lung sections of rats exposed to 500 μg NPs evaluated at D21 after elastase administration. (**A**) Representative histological images of staining for MMP-12 (magnification x20, left column) and CD68 (magnification x20, middle column, magnification x40, right column). Arrows point to macrophages containing NPs. (**B**) Quantification of MMP-12-positive cells reported to the total number of cells. (**C**) Quantification of CD68-positive cells reported to the total number of cells. *: *p* < 0.05 vs S-BSA. n = 4. Abbreviations are the same as in Figure1. Box plots show median and interquartile range (25^th^ and 75^th^ percentiles).

To verify whether the increase in MMP-12 mRNA level was also observed at the protein level**,** we analyzed the protein expression of MMP-12 at D21 by immunohistochemical staining. With saline treatment, staining for MMP-12 protein was greater with CB NPs than BSA exposure (*p* < 0.05, Figure6A, B). Moreover, staining was greater with elastase than saline treatment, regardless of NP exposure.

Because MMP-12-positive cells showed morphologic features of macrophages, we verified this by performing CD68 immunostaining in a serial slide compared to the one in which MMP-12 was detected. Figure6C shows results for CD68 staining similar to that for MMP-12 staining. Besides, most cells expressing MMP-12 also expressed CD68, which strongly suggests that cells expressing MMP-12 were macrophages. Stained cells were located in the alveolar lumen and in the perivascular and peribronchial infiltrates (Figure6A). Additionally, NPs aggregates were observed in some CD68 positive cells.

## Discussion

The main results of our study are that: *1)* TiO_2_ NPs did not induce inflammation *per se* and did not aggravate elastase-induced pulmonary inflammation and emphysema, except for an increase in elastase-induced MMP-12 mRNA expression in the lung, and *2)* CB NPs induced perivascular/peribronchial infiltration, increased HO-1 mRNA expression in the lung and increased MMP-12 mRNA and protein expression in alveolar macrophages without inducing emphysema. CB NPs aggravated elastase-induced perivascular/peribronchial inflammation but not emphysema. These results demonstrate that TiO_2_ or CB NPs did not induce and did not aggravate elastase-induced emphysema. However, CB NPs induced a lung inflammatory and protease response close to that induced by elastase and, notably, macrophage MMP-12 expression.

### Methodological considerations

We decided to focus this study on the evaluation of NPs at doses that were relevant in terms of human exposure. We reasoned in terms of the current time-weighted average concentrations (TWA) recommendations for workers exposed to TiO_2_ (http://www.cdc.gov/niosh/review/public/tio2/pdfs/TIO2Draft.pdf). These values are between 1.5 and 15 mg/m^3^ for a single shift of work (Table1 of the above referenced document). In the present study, we used 0.4 and 2 mg/kg of TiO_2_ NPs, corresponding to a total administered-mass of 100 and 500 μg per rat, for a 250 g rat. For a 70 kg human, this concentration would lead to the total administration of 28 and 140 mg. For a worker inhaling 10 m^3^ per workday, it implies TiO_2_ concentrations exposure of 2.8 and 14 mg/m^3^, which are in the 2 extremes of the range of TWA values. Of course, we are aware that only a fraction of this inhaled dust reaches and remains in the lung. However, it is known that TiO_2_ particles have a long retention half-time (between 170 and 500 days for the fine and ultra-fine TiO_2_ particles respectively [31]). Therefore, the calculation of the total administered dose must take into account the duration between the day of NP administration and the day the animals are sacrificed. In our study, we observed a 14 days period between administration and sacrifice. Therefore, the dose initially administrated should be divided by 14, which leads to values well below TWA values.

A potential criticism of this study is the respiratory delivery of NPs by a single intratracheal instillation instead of aerosol exposure. We acknowledge that aerosol exposure better reflects human exposure than intratracheal instillation. However, intratracheal instillation is a validated method of respiratory NPs delivery (see for example [32,33]). Moreover, given the long retention half time of NPs [31], a single instillation is well adapted to reproduce continuous exposure during short-term experimental periods like in the present study. It has to be noted that we used BSA to disperse the NPs in solution and this procedure could influence the biological effects of the NPs, as demonstrated by Val and colleagues in bronchial epithelial cells exposed to CB and TiO_2_ NPs [17]. However, other investigators did not find such interference with investigation of different types of carbon nanotubes [23]. This aspect should deserve further investigation.

Another potential criticism of this study is the utilization of the elastase-induced pulmonary emphysema model to analyze emphysema induced by cigarette smoke exposure, as occurs in humans. We decided not to use the cigarette smoke model because cigarette smoke contains NPs [34] that could prevent from examining the only effect of CB and TiO_2_ NPs on emphysema. We think that the elastase model was appropriate for the present study for the following reasons. First, animals receiving a bolus of exogenous elastase into the lung exhibit lung damage consistent with emphysema, which is progressive [21], and involves many of the main pathophysiologic changes observed in cigarette smoke-induced emphysema in humans and animals, i.e. inflammation, oxidative stress [35], alveolar cell apoptosis [20][21], and MMP-12 induction [21]. Second, elastase-induced emphysema can be modulated by different conditions (i.e. IL-6 or Nrf2 knocking down [21,35]), showing that it’s a dynamic process, susceptible to be modulated by exposure to NPs, which does not result only from initial tissue destruction by exogenous elastase. Moreover, we took care of using a dose of elastase inducing a degree of emphysema similar to that observed after cigarette smoke exposure (near 30% increase in mean linear intercept [36]). Finally, the expression of major actors of cigarette smoke-induced emphysema, such as HO-1, MMP-12 and IL-1β was increased in our elastase-instilled animals, supporting the relevance of the model.

Emphysema was quantified by the measurement of the mean chord length, which is a standard stereological method to quantify alveolar surface area [27,28,37,38]. However, care must be taken when using this method because it can be fraught with a number of pitfalls [27,28]. First, intercept lengths measured on microscopic slides are primarily determined by the inflation level at which the specimen has been held during tissue preparation. Therefore, in order to ensure the accurate comparison of the different groups, particular attention was paid to fix the lungs at a similar transpleural pressure (25 cmH_2_O for 3 h) before holding them in 4% paraformaldehyde. Second, we ensured that sampling of intercepts avoid edge effects that cause underrepresentation of very long intercepts as they occur, when the test line lies in the direction of alveolar ducts. Finally, care was taken to avoid any particle contained in the air space image (a speck of dust for example) that may intersect the scan line and thus cut an intercept in half so that two short chords are recorded instead of one longer one. This point is particularly important in animals exposed to NPs, since one can consider that isolated NPs in alveolar lumen could behave like a speck of dust, leading thus to underestimation of emphysema in these animals. However, no isolated NPs were observed, and thus this possibility can be ruled out.

### Results discussion

We compared NPs that had similar sizes, but different specific surface areas, since specific surface area was 3 times greater for CB than for TiO_2_ NPs. *In vivo* and *in vitro* studies demonstrated that the surface area of NPs could be a main determinant of their pro-inflammatory effects [16,39-42]. This parameter and/or a different surface reactivity, related to the chemical nature of the NPs, could explain the difference in their inflammatory effects in the present study. It has been demonstrated that TiO_2_ and CB NPs elicited distinct apoptotic pathways [43], which could also impact their effects on the animals. However, no morphological sign of apoptosis was observed in the rat lungs. In contrast to TiO_2_ NPs, CB NPs induced inflammation and expression of the oxidative stress marker HO-1 and the protease MMP-12. Using the same NPs as we used, Hussain and coworkers [44] showed that the reactivity of CB NPs was higher than that of TiO_2_ NPs, as evaluated by the production of reactive oxygen species in acellular conditions. The increase in HO-1 expression that we observed supports an oxidative effect of CB NPs [45,46], which has been already described in bronchial epithelial cells [43], and could be responsible for the inflammatory effect of CB NPs [47]. Although we did not find an increase in inflammatory cells in BAL, we observed clear inflammatory histological alterations (perivascular and peribronchial infiltration of neutrophils, lymphocytes and macrophages), as observed previously with other particles [44,48]. Macrophages engulfed CB NPs, apparent as pigmentation in the histological sections. In addition to attracting macrophages to the lung, CB NPs activated these cells, as revealed by their increased MMP-12 protein expression detected by immunohistochemistry. We and others [48,49] showed that different particles, such Paris Metro or atmospheric particulate matter (PM 10), could induce MMP-12 expression in macrophages. The present results provide the first demonstration of an increase in MMP-12 expression by manufactured NPs, both at the mRNA and protein levels.

Although we analyzed MMP-12 protein expression and its localization, we did not measure its activity. However, our results showing MMP-12 protein expression in macrophages are compatible with the presence of an active enzyme. Indeed, Cobos-Correa and coworkers [49] demonstrated in mice intratracheally instilled with PM 10 particles that active MMP-12 is bound to the membrane of alveolar macrophages. The increase in MMP-12 expression after a single exposure CB NPs could have important pathophysiological consequences because this protease plays a critical role in emphysema and COPD [50,51]. Continuous exposure to relevant doses of CB NPs could be consistent with a continuous degradation of pulmonary elastin, which could lead to a progressive and chronic degradation of lung function, as observed in smokers developing COPD [51]. Oxidative stress, revealed by the increased HO-1 expression, could explain MMP-12 induction after CB NPs administration [52].

TiO_2_ NPs did not induce inflammation or protease expression *per se* but, rather, potentiated MMP-12 mRNA induction by elastase. This result is in line with data from Hussain and coworkers [44] showing that TiO_2_ NPs, at a dose similar to ours, potentiated toluene diisocyanate-induced allergic inflammation in mice without inducing inflammation *per se.* However, the relevance of the potentiation of elastase-induced MMP-12 mRNA expression by TiO_2_ is questionable, because we were unable to detect a parallel increase in protein expression. Moreover, this phenomenon was observed in the absence of a parallel increase in HO-1 and IL-1β expression, which suggests that the effect of TiO_2_ NPs on MMP-12 expression is independent of oxidative stress or IL-1β induction. Further experiments are needed to better understand this phenomenon.

CB NPs were unable to potentiate elastase-induced emphysema, although they induced inflammation and MMP-12 expression *per se* and potentiated elastase-induced perivascular/peribronchial infiltration. This result agrees with data reported by Inoue and coworkers [18] in the elastase-induced emphysema model showing that CB NPs with a specific surface area and a diameter similar to our NPs, and at a dose similar to that used in our study, also induced inflammation *per se* and potentiated elastase-induced inflammation but not emphysema. Inoue and coworkers [18] administered CB NPs simulatenously with elastase whereas we administered NPs 1 week after elastase to assess their effects on the development of already constituted but still progressing emphysema [21]. The similar absence of potentiation of emphysema in the 2 studies shows that the timing of CB NPs administration is not a major determinant of this phenomenon. One car argue that the relatively short period elapsed after CB NPs could be insufficient to allow emphysema potentiation. Although we cannot exclude this hypothesis, other interventions such as administration of macrophage-colony stimulating factor, in a similar time frame than in the present study, was able to aggravate elastase-induced emphysema [53]. Interestingly, the potentiation of elastase-induced inflammation by CB NPs is in contrast with a reduced inflammatory response to bacterial infection in elastase-instilled animals [54,55], suggesting a specific effect of NPs in this model.

## Conclusion

This study demonstrates an absence of induction and aggravation of elastase-induced emphysema with the administration of relevant doses of TiO_2_ or CB NPs. This result shows that not all the exposures to different NPs are associated with adverse health effects. However, CB NPs *per se* aggravated elastase-induced histological inflammation and increased expression of MMP-12, a major protease involved in elastin breakdown. Further studies will be needed to examine the implications of these findings.

## Abbreviations

BAL: Bronchoalveolar lavage; BET: Brunauer Emmett Teller; BSA: Bovine serum albumin; CB: Carbon black; COPD: Chronic obstructive pulmonary disease; HO-1: Heme oxygenase-1; HPRT: Hypoxanthine phosphoribosyltransferase; IL-1β: Interleukin 1β; IQR: Interquartile range; LAL: Limulus Amebocyte Lysate; MCP-1: Monocyte chemotactic protein 1; MIP-2: Macrophage inflammatory protein 2; MMP: Matrix metalloproteinase; NPs: Nanoparticles; PSD : Particle Size Distribution function; RT-qPCR: Real-Time quantitative PCR; TiO_2_: Titanium dioxide; TEM: Transmission electronic microscopy.

## Competing interests

The authors declare that they have no competing interests.

## Authors’ contribution

SL, GL and JB designed the study. LA, EB and ASD characterized the NP. AR, FR and GL conducted the animal’s instillations experiments. AR, LA and MD performed the biological assays. JTVN helped with the histological analysis of inflammation. LA, and JB drafted the manuscript, and JCP and SL helped with the final version. All authors read and approved the final manuscript.

## Pre-publication history

The pre-publication history for this paper can be accessed here:

http://www.biomedcentral.com/1471-2466/12/38/prepub
